# MDT-28/PLIN-1 mediates lipid droplet-microtubule interaction via DLC-1 in *Caenorhabditis elegans*

**DOI:** 10.1038/s41598-019-51399-z

**Published:** 2019-10-17

**Authors:** Kang Xie, Peng Zhang, Huimin Na, Yangli Liu, Hong Zhang, Pingsheng Liu

**Affiliations:** 1 National Laboratory of Biomacromolecules, CAS Center for Excellence in Biomacromolecules, Institute of Biophysics, Chinese Academy of Sciences, Beijing, 100101 China; 2 University of Chinese Academy of Sciences, Beijing, 100049 China; 3 Research Division, Joslin Diabetes Center, One Joslin Place, Boston, MA 02215 USA; 4 Department of Genetics and Harvard Stem Cell Institute, Harvard Medical School, Boston, MA 02215 USA; 5 Program in Systems Biology and Program in Molecular Medicine, University of Massachusetts Medical School, Worcester, MA USA

**Keywords:** Organelles, Multivesicular bodies, Biochemistry

## Abstract

Ectopic lipid accumulation in lipid droplets (LD) has been linked to many metabolic diseases. In this study, DHS-3::GFP was used as a LD marker in *C*. *elegans* and a forward genetic screen was carried out to find novel LD regulators. There were 140 mutant alleles identified which were divided into four phenotypic categories: enlarged, aggregated, aggregated and small, and decreased. After genetic mapping, mutations in three known LD regulatory genes (*maoc-1*, *dhs-28*, *daf-22*) and a peroxisome-related gene (*acox-3*) were found to enlarge LDs, demonstrating the reliability of using DHS-3 as a living marker. In the screen, the cytoskeleton protein *C27H5*.*2* was found to be involved in LD aggregation, as was the LD resident/structure-like protein, MDT-28/PLIN-1. Using yeast two-hybrid screening and pull-down assays, MDT-28/PLIN-1 was found to bind to DLC-1 (dynein light chain). Fluorescence imaging confirmed that MDT-28/PLIN-1 mediated the interaction between DHS-3 labeled LDs and DLC-1 labeled microtubules. Furthermore, MDT-28/PLIN-1 was directly bound to DLC-1 through its amino acids 1–210 and 275–415. Taken together, our results suggest that MDT-28/PLIN-1 is involved in the regulation of LD distribution through its interaction with microtubule-related proteins.

## Introduction

The lipid droplet (LD) is a cellular organelle found in nearly all species ranging from bacteria to humans^1–3^. It consists of a neutral lipid core surrounded by a phospholipid monolayer membrane containing characteristic proteins. LDs have been found to play many important roles, including lipid storage and transportation, metabolic regulation, and serving as a source of membrane lipid precursors. Abnormal LD dynamics are related to many human diseases including diabetes, lipodystrophies, fatty liver disease, and cardiovascular disorders. This connection with human health is an important factor driving the attention of researchers into the dynamics of LDs^4–6^.

The major LD-proteins perilipin^7^, adipose differentiation-related protein (ADRP)^8^, tail-interacting protein of 47 kDa (TIP47)^9^, S3-12^10^, and OXPAT^11^ were identified as members of the PAT family proteins due to their common PAT domain. Later, they were reclassified as the perilipin (PLIN) family, members 1 through 5^12^. Although they were originally found in mammals, comparative sequence analysis has identified two homologues in *Drosophila* and one in slime molds. Among these proteins, only perilipin/PLIN-1 and ADRP/PLIN-2 can be considered as LD resident proteins since their cellular distribution is largely restricted to LDs^13^. The other family members are more broadly distributed in cells^14^.

Over the past two decades, myriad investigations using these two LD resident proteins as markers have revealed that LD is an active organelle with surprising complexity. Important contributions have been made through the isolation of LDs followed by proteomic analysis. LD-associated proteins have been identified from many organisms and cell types using this approach^1,15^. Many of the LD-associated proteins have been identified to be involved in lipid metabolism, intracellular trafficking, signaling, RNA metabolism, and cytoskeletal organization^16–22^. The broad range of activities of these proteins suggests that LDs are actively engaged in many cellular functions beyond static lipid storage. Some of these recently identified roles have been tested and verified, thus providing new clues regarding the mechanisms underlying many metabolic diseases^6^.

However, the mammalian system, in which most work on LDs has been conducted, has limited tools for screening or genetic manipulation. Therefore, *C*. *elegans* is an attractive system to accelerate discovery due to its genetic tractability and an intimate connection between lipid metabolism, reproduction, and lifespan^23^. To find the LD resident proteins, LDs were isolated from *C*. *elegans* and two major/resident LD proteins, DHS-3 and MDT-28/PLIN-1, were identified using proteomic analysis and molecular biology^13,24^. By sequence analysis, DHS-3 belongs to the HSD family and MDT-28/PLIN-1 is similar to a mammalian mediator protein MED-28 (Mediator of RNA polymerase II transcription subunit 28, Q9H204)^13^ (Fig. S1). Interestingly, MDT-28/PLIN-1 contains an N-terminal domain which has some sequence similarity to the PAT domain^25^. Using fluorescent fusion proteins in a tissue distribution study, DHS-3 was found to mainly locate in intestinal cells while MDT-28/PLIN-1 was distributed in the hypodermis, muscle and intestine^13^.

Using DHS-3 as LD markers in *C*. *elegans*, we identified mutant strains with altered LD morphology, especially the molecular mechanisms underlying LD distribution. After analyzing the phenotype of some candidate genes, we found that the deletion of MDT-28/PLIN-1 or C27H5.2 can cause aggregation of LDs. To further investigate the mechanism of MDT-28/PLIN-1 mutation leading to LD aggregation, we explored it from two lines. (1) a forward genetic screen with DHS-3 as a reporter was carried out. Among the candidate genes, mutation in *C27H5*.*2* exhibits a phenotype similar to MDT-28/PLIN-1. (2) Found the binding protein of MDT-28/PLIN-1 by yeast two-hybrid. By results of the above experiments, we found several genes involved in LD distribution including cytoskeleton-associated protein. In recent years, a growing number of studies have reported interactions between LDs and microtubules^26^, especially during *Drosophila* embryogenesis^27–31^. However, the LD-associated protein(s) which mediate LD-microtubule interaction and the detailed mechanisms governing LD distribution in *C*. *elegans* are unknown.

Microtubules are polarized filaments that provide the track for the movement of cargo organelles via motor proteins such as dynein. The components of motor proteins are well understood. However, it is unclear how motor proteins are regulated to traffic specific organelles to their destination with a high degree of spatial and temporal precision^30,32^. In this study, MDT-28/PLIN-1, as the LD resident/structure-like protein, was a putative binding partner of the dynein light chain (DLC-1) in *C. elegans*. Our studies provide an insight into the interaction between LDs and microtubules and the regulation of LD distribution.

## Results

### Mutations of *C27H5*.*2* induce lipid droplet aggregation phenotypes

Our previous work demonstrated that MDT-28/PLIN-1/MED28 and DHS-3/17βHSD11 are the most abundant LD resident proteins in *C*. *elegans*^13^. Interestingly, MDT-28/PLIN-1 deletion induces LD aggregation in the L4 larval stage^13^, slightly reduced the brood size (Fig. S2A), but the underlying mechanism is not understood. To better show the phenotype of LD aggregation, we conducted a forward genetic screen to search for other genes inducing LD aggregation to better understand the role of MDT-28/PLIN-1. We used a single copy *Pvha-6::dhs-3::GFP* strain and a forward genetic screen to search for other proteins which influence LD morphology or cellular distribution.

**Figure 1:**
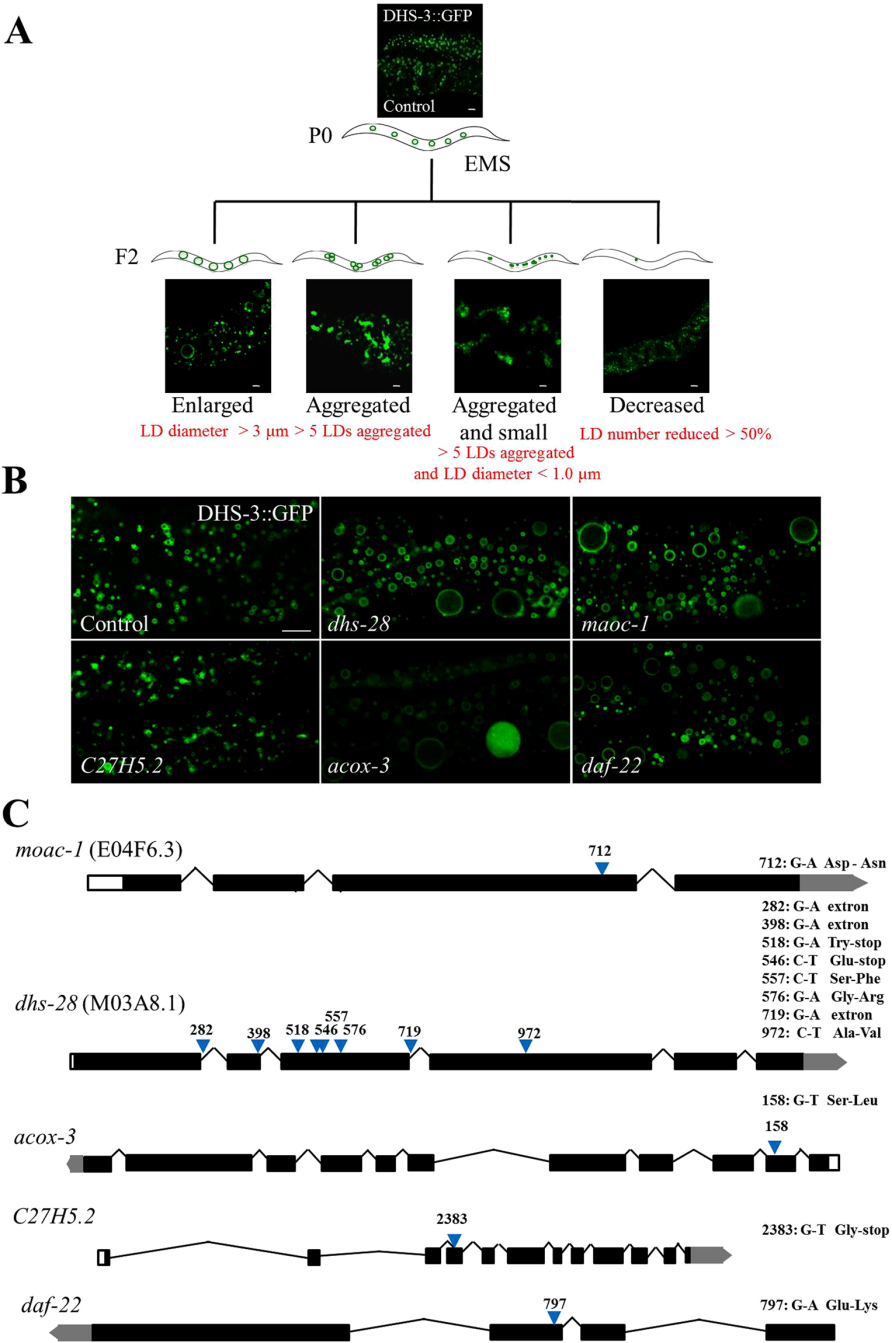
EMS screening using DHS-3::GFP as a LD marker. (**A**) Schematic representation of the forward genetic screen. Visualization of LDs using the marker DHS-3::GFP in larval L4 stage animal. The four groups of LD morphologies: enlarged, aggregated, aggregated and small, and decreased. Scale Bar, 5 μm. (**B**) Visualization of LDs using the marker DHS-3::GFP in *maoc-1*, *dhs-28*, *daf-22*, *acox-3* and *C27H5*.*2* mutants. Scale Bar, 5 μm. (**C**) Schematic representation of the gene structures and mutation sites of *maoc-1*, *dhs-28*, *daf-22*, *acox-3* and *C27H5*.*2*. “extron” is defined as a mutation in the first intron nucleotide adjacent to upstream exon which can affect splicing.

The *Pvha-6::dhs-3::GFP* strain was mutagenized with ethyl methane sulfonate (EMS) and 140 mutants with altered LD morphologies were identified. The mutants were grouped into four categories based on LD number, size, and dispersion: enlarged, aggregated, aggregated and small, and decreased (Fig. 1A). The ‘enlarged’ phenotype was defined as LDs larger than 3 μm (Under normal circumstances, the average diameter of the LDs in the tail of the nematode is 1.5–1.8 μm). Animals with an ‘aggregated’ phenotype had at least 5 LDs clustered together. The phenotype ‘aggregated and small’ contained clusters of 5 LDs with a diameter less than 1.0 μm. The ‘decreased’ phenotype had a reduced overall number of LDs (The number of LDs is significantly reduced, reducing by more than 50%). After backcrossing the N2, we used genetic mapping to identify four genes which induced the enlarged phenotype. Three are known genes (*maoc-1*, *dhs-28*, *daf-22*) and one is newly identified (*acox-3*) in LD biology (Fig. 1B). RNAi feeding experiments in wild type animals confirmed the phenotype of the mutants (Fig. S2B).

Both *dhs-28* and *moac-1* encode orthologs of human 17-β-hydroxysteroid dehydrogenase 4 (HSD17B4) (Fig. 1B,C). *daf-22* encodes the *C*. *elegans* ortholog of human sterol carrier protein SCP2, and *acox-3* is an ortholog of human acyl-CoA oxidase 3 (ACOX3, pristanoyl)^33^. Both ACOX3 and SCP2 are related to peroxisomal fatty acid beta-oxidation. Previous studies in *C*. *elegans* have shown that knock down or knockout of *maoc-1*, *dhs-28*, or *daf-22* leads to the enlarged LDs^34^. The *acox-3* protein is in the same pathway with *maoc-1*, *dhs-28* and *daf-22*^34^. These results demonstrate the utility of DHS-3 as a marker for identifying LD phenotypes.

Among the mutants with the aggregated phenotype, we identified *C27H5*.*2*, which interacts with UNC-83^35^, a component of the dynein complex. Mutation of *C27H5*.*2* induced LD aggregation in *C*. *elegans*. (Fig. 1B)

### Mutation of *C27H5*.*2* induced LD aggregation similar to the *mdt-28*(*tm1704*) phenotype

**Figure 2:**
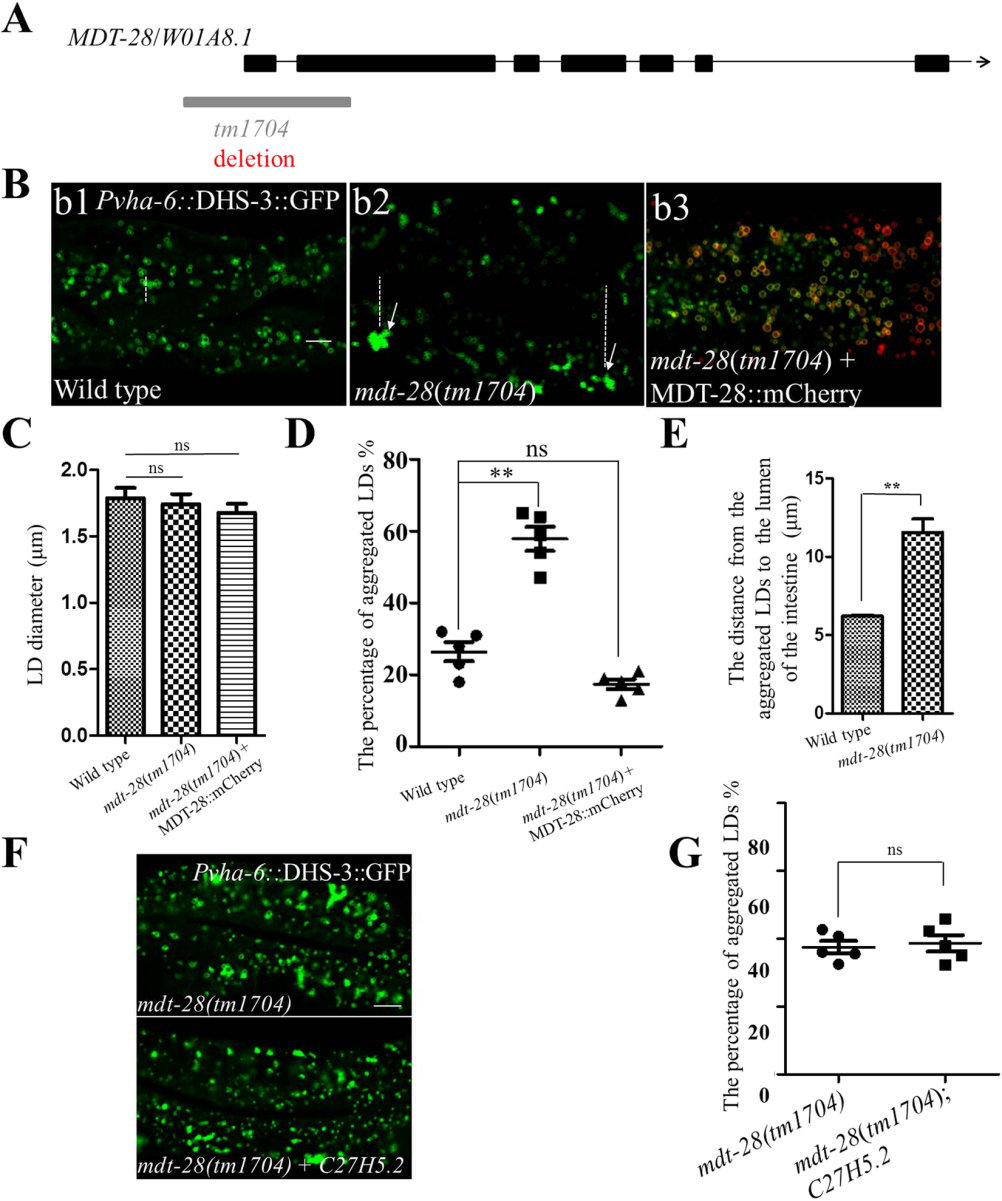
Mutations of *C27H5*.*2* and *mdt-28*/*plin-1* induce similar lipid droplet aggregation phenotypes. (**A**) Schematic representation of the gene structures and mutation sites of *mdt-28*. (**B**) Fluorescence 2D micrographs of LDs in the intestine, Scale Bar, 5 μm. (b1) Fluorescence 2D micrographs of *Pvha-6::*DHS-3::GFP in a larval L4 stage animal. (b2) Visualization of LDs using the marker *Pvha-6::*DHS-3::GFP in the *mdt-28*(*tm1704*) mutant. The white arrows point to aggregated LDs. Dotted line represents the distance from aggregated LDs to intestinal axis. (b3) As in (b2), but with the *mdt-28*(*tm1704*) mutant animal carrying a recue transgene [*mdt-28p::mdt-28::mCherry*]. (**C**) Quantification of the LD diameter (B). Data represent mean ± SEM (n = 5 for each independent experiment, ns, no significance, one-way ANOVA). (**D**) Quantification of the percentage of aggregated LDs (B). Data represent mean ± SEM (n = 5 for each independent experiment, **P < 0.01, ns, no significance, one-way ANOVA). (**E**) The distance from the aggregated LDs to the lumen of the intestine was quantified (B). Data represent mean ± SEM (n = 5 for each independent experiment, **P < 0.01, student *t*-test). (**F**) Images of LDs using the marker *Pvha-6::DHS-3::GFP* in *mdt-28*(*tm1704*) worms and *mdt-28*(*tm1704*)*; C27H5*.*2* worms, Scale Bar, 5 μm. (**G**) Quantification of the percentage of aggregated LDs (F). Data represent mean ± SEM (n = 5 for each independent experiment, ns, no significance, P > 0.05, student *t*-test).

To further investigate the mechanism of LD aggregation, other genes were introduced in this study, such as *mdt-28*/*plin-1*. To show the phenotype of MDT-28, *tm1704* (a loss-of function of MDT-28/PLIN-1) was used to study the phenotype of *mdt-28* mutants (Fig. 2A–E). As previous work reported, MDT-28/PLIN-1 deletion induces LD aggregation in the L4 larval stage in *C*. *elegans*. We performed additional analysis of the mutants and found that 69% of LDs in *mdt-28*(*tm1704*) larval L4 stage worms were aggregated compared with under 20% for wild type (Fig. 2B,D). When MDT-28/PLIN-1 was re-introduced into the *mdt-28*(*tm1704*) mutants, the degree of intestinal LD aggregation was reduced, demonstrating a rescue of normal LD morphology (Figure 2Bb3 and 2D). To better show the phenotype of LD aggregation, we used the LSM880 with airyscan for 3D imaging (Fig. S3A). The degree of aggregation of the LDs was similar to the 2D result (Fig. S3B). Analysis of 2D imaging results found that aggregated LDs were distributed at the edge of the distal intestine in *mdt-28*(*tm1704*) mutants (Figs 2Bb2,E and S3Aa2).

Through analysis of phenotypes, mutation of *C27H5*.*2* induced LD aggregation similar to the *mdt-28*(*tm1704*) phenotype. Compared with *mdt-28*(*tm1704*) mutants, *C27H5*.*2*; *mdt-28*(*tm1704*) mutants did not increase the percentage of aggregated LDs (Fig. 2F,G), suggesting they belong to a common mechanism. Since the distribution of LDs is likely related to their role in lipid transport and the mechanisms governing this distribution are not well understood, we next focused on the relationship between LDs and cytoskeleton.

### Nocodazole reduces LD size in the wild type and enhances LD aggregation in *C27H5*.*2* mutants

**Figure 3:**
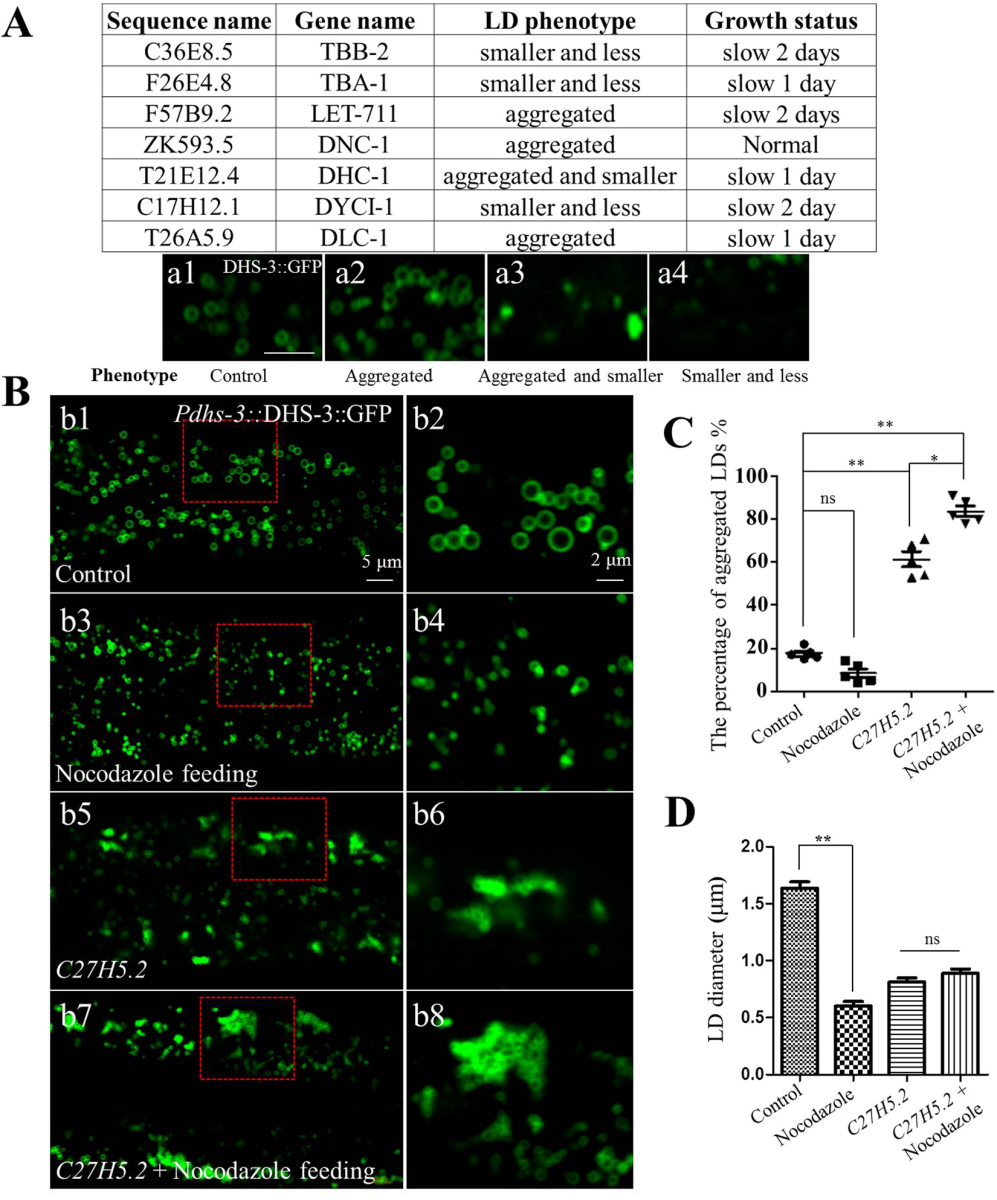
Nocodazole reduces LD size in the wild type and enhances LD aggregation in C27H5.2 mutants. (**A**) The genes identified by RNAi screening which affect LD phenotype and growth status. Visualization of LDs using the marker *Pdhs-3*::DHS-3::GFP in larval L4 stage animals (a1,a2,a3,a4). Scale Bar, 2 μm. (**B**) Nocodazole affected the morphology of LDs. (b1) Visualization of LDs using the marker *Pdhs-3*::DHS-3::GFP in L4 stage animals. Scale Bar, 5 μm. (b3) Visualization of LDs using the marker *Pdhs-3*::DHS-3::GFP after feeding worms with 5 µM Nocodazole from L1 to L4 stage. (b5) Visualization of LDs using the marker *Pdhs-3*::DHS-3::GFP in *C27H5*.*2* mutants. (b7) Visualization of LDs using the marker *Pdhs-3*::DHS-3::GFP after feeding *C27H5*.*2* mutants with 5 µM Nocodazole from L1 to L4 stage. (b2,b4,b6,b8) are the enlarged picture of (b1,b3,b5,b7). Scale Bar, 2 μm. The percentage of LD aggregation (**C**) and the diameter of LDs (**D**) for (b1,b3,b5,b7) were quantified. Data represent mean ± SEM (n = 5 for each independent experiment, **P < 0.01, ns, no significant, two-way ANOVA).

We performed an RNAi screen of genes related to the cytoskeleton to search for knockdowns which induce a LD phenotype. We selected 246 genes to cast a wide net for candidate proteins (Table S1). Knock down of many microtubule genes resulted in unhealthy worms which grew slowly and were sterile (Table S1). We selected several candidate genes which affected LD phenotype for further study (Fig. 3A). By knocking down 7 representative genes, two LD phenotypes were observed: decreased (reduced number of LD) (*tbb-2*, *tba-1*, or *dyci-1*) and aggregated (increased LD aggregation) (*let-711*, *dlc-1*, *dnc-1*, or *dhc-1*) (Fig. 3A).

Next, the microtubule polymerization inhibitor nocodazole was used to confirm the role of the microtubule in LD morphology. Wild type worms fed with nocodazole had small LDs (Fig. 3Bb1–4,D) and the LD aggregation in *C27H5*.*2* mutants was enhanced when they were fed with nocodazole (Fig. 3Bb5–b8,C). Therefore, the cytoskeleton is clearly involved in LD morphology and distribution as demonstrated through genetic and chemical manipulation.

### MDT-28/PLIN-1 interacts with dynein light chain 1

**Figure 4:**
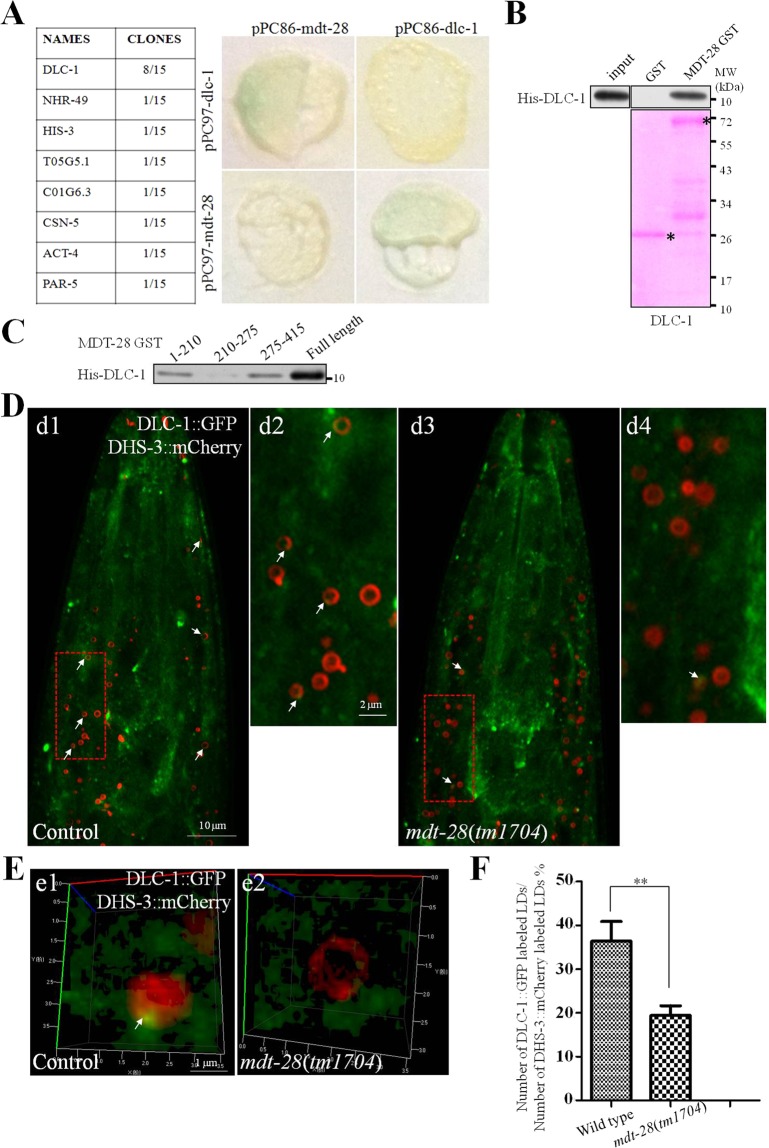
MDT-28/PLIN-1 interacts with DLC-1. (**A**) Detection of the MDT-28/DLC-1 interaction using the yeast two-hybrid (Y2H) assay. Yeast cells co-transformed with pPC97-MDT-28 and a pPC86**-***C*. *elegans* library were placed on high-stringency selective medium plates (SD/Trp-Leu-His-Ade) to find positive interactions. Staining with β-gal confirmed DLC-1 in the screen. (**B**) In a pull-down assay, MDT-28-GST immobilized on glutathione Sepharose beads specifically bound to His-DLC-1 as detected by Western blot with anti-His. (**C**) Truncation mutations of MDT-28 were made based on hydrophobicity and predicted alpha helices. His-DLC-1 binding by MDT-28 (1–210, 211–274, 275–415)-GST fusion proteins was detected by Western blot with anti-His. (**D**) Fluorescence 2D micrographs of DHS-3::mCherry labeled LDs (red) interacting with DLC-1 (green). (d1) Visualization of DLC-1::GFP in the wild type larval L4 stage animal. The LD marker DHS-3::mCherry was used in the head and merged with DLC-1::GFP. Scale Bar, 10 μm. (d2) is the enlarged pictures of (d1). Scale Bar, 2 μm. (d3) As in (d1), but in a *mdt-28*(*tm1704*) mutant. (d4) is the enlarged pictures of (d3). The white arrows point to the co-localization sites. (**E**) (e1) Fluorescence 3D micrographs of DHS-3::mCherry and DLC-1::GFP in a larval L4 stage animal. Scale Bar, 1 μm. (e2) As in (e1), but in the *mdt-28*(*tm1704*) mutant. The white arrows point to the co-localization sites. (**F**) Number of DLC-1::GFP labeled LDs/Number of DHS-3::mCherry labeled LDs for (D). Data represent mean ± SEM (n = 5 for each independent experiment, **P < 0.01, student *t*-test).

To further explore the cause of LD aggregation in the *mdt-28* mutants, a yeast two hybrid (Y2H) assay was conducted to search for MDT-28/PLIN-1’s binding partners. Among the obtained colonies, dynein light chain 1 (DLC-1) was the most confident hit, comprising 8 out the 15 clones (8 of the 15 obtained clones were DLC-1) (Fig. 4A). Galactosidase activity was confirmed in the DLC-1 clones. To confirm the interaction between DLC-1 and MDT-28/PLIN-1, the plasmids pPC86-*dlc-1* and pPC97*-mdt-28* were co-transformed into yeast *ma203*, and the yeast was plated on selective media. The results verified that MDT-28/PLIN-1 interacted with DLC-1 (Fig. 4A). Next, a pull-down assay was carried out *in vitro*. MDT-28-GST, His-DLC-1, and His-LGG-1 were independently purified, and MDT-28-GST was immobilized on glutathione sepharose beads. MDT-28-GST beads were mixed and incubated with either His-DLC-1 or the control His-LGG-1. The beads were washed multiple times and were recovered. LGG-1 was not bound to MDT-28/PLIN-1 (Fig. S5A), but His-DLC-1 was specifically bound to the glutathione beads as shown by Western blotting with anti-His antibody (Fig. 4B).

To determine the region of MDT-28/PLIN-1 involved in DLC-1 binding, GST fusion proteins of MDT-28/PLIN-1 truncation mutants (1-210, 211-274, 275-415) were constructed, with truncations based on hydrophobicity and predicted alpha helices. The 1-210-GST and 275-415-GST proteins were found to bind His-DLC-1 using the pull-down assay (Fig. 4C). The results demonstrate that both N-terminal and C-terminal domains of MDT-28/PLIN-1 are sufficient for inter-molecular interaction with His-DLC-1.

To study the interactions between DLC-1 and MDT-28/PLIN-1 *in vivo*, a transgenic line, *Pdlc-1::dlc-1::GFP*, was generated and examined by fluorescence microscopy. DLC-1 was expressed in the intestine, muscle, hypodermis, and the rectal valve cells, and unidentified cells in the head (Fig. S4). This distribution was consistent with previous observations^36^. We next constructed *Pmdt-28::mdt-28::mCherry*; *Pdlc-1::dlc-1::GFP* double transgenic worms and examined them using the LSM880 microscope. However, the DLC-1::GFP fluorescence was too weak and the intestinal cell LDs too numerous to detect interactions. Therefore, we analyzed the double transgenic worm DHS-3::mCherry; {*CasIs581 [dlc-1::GFP ki*]}^37^. We selected the worm’s head for analysis because of higher DLC-1::GFP expression and relatively low number of LDs, which facilitated the observation of interactions between LDs and microtubules. In the wild type, DHS-3::mCherry-labeled LDs were partially co-localized with DLC-1::GFP and 37% of LDs were labeled by DLC-1::GFP signal (Fig. 4D). However, co-localization was reduced in the *mdt-28*(*tm1704*) mutant (Fig. 4F). We also analyzed the co-localization of DHC-1::GFP, and the control protein GFP::LGG-1, with DHS-3::mCherry. There was no co-localization of DHS-3::mCherry-labeled LDs with GFP::LGG-1 in the wild type or *mdt-28*(*tm1704*) mutant, but DHC-1::GFP was co-localized with DHS-3::mCherry-labeled LDs in the wild type (Fig. S5B). We performed additional co-localization experiments using the LSM880 microscope for 3D imaging (Fig. 4E). In the wild type, DHS-3::mCherry-labeled LDs were co-localized with DLC-1::GFP, but this association was reduced in the *mdt-28*(*tm1704*) mutant. These results suggest that MDT-28/PLIN-1 mediates the interaction between DHS-3 labeled LDs and DLC-1 labeled microtubules. To further confirm this result, we used the super resolution structured illumination microscopy (SIM) to observe the DLC-1::GFP labeled microtubule pattern and MDT-28/PLIN-1 labeled LD pattern. There is a technical difficulty, DLC-1::GFP labeled microtubule is hard to detect in hypodermis and intestine while LDs existed in the main tissues, but it is easy to be detected in body wall muscles while LDs existed less in the muscle. We used the SIM to capture LDs and microtubule dynamic structure, few lipid droplets could be captured to bind to DLC-1::GFP, the enlarged pictures could be used as a evidence that DLC-1 co-localized with MDT-28/PLIN-1 in muscle (Fig. S6).

### *dlc-1* RNAi slightly enhances lipid droplet aggregation in *mdt-28*(*tm1704*) mutants

**Figure 5:**
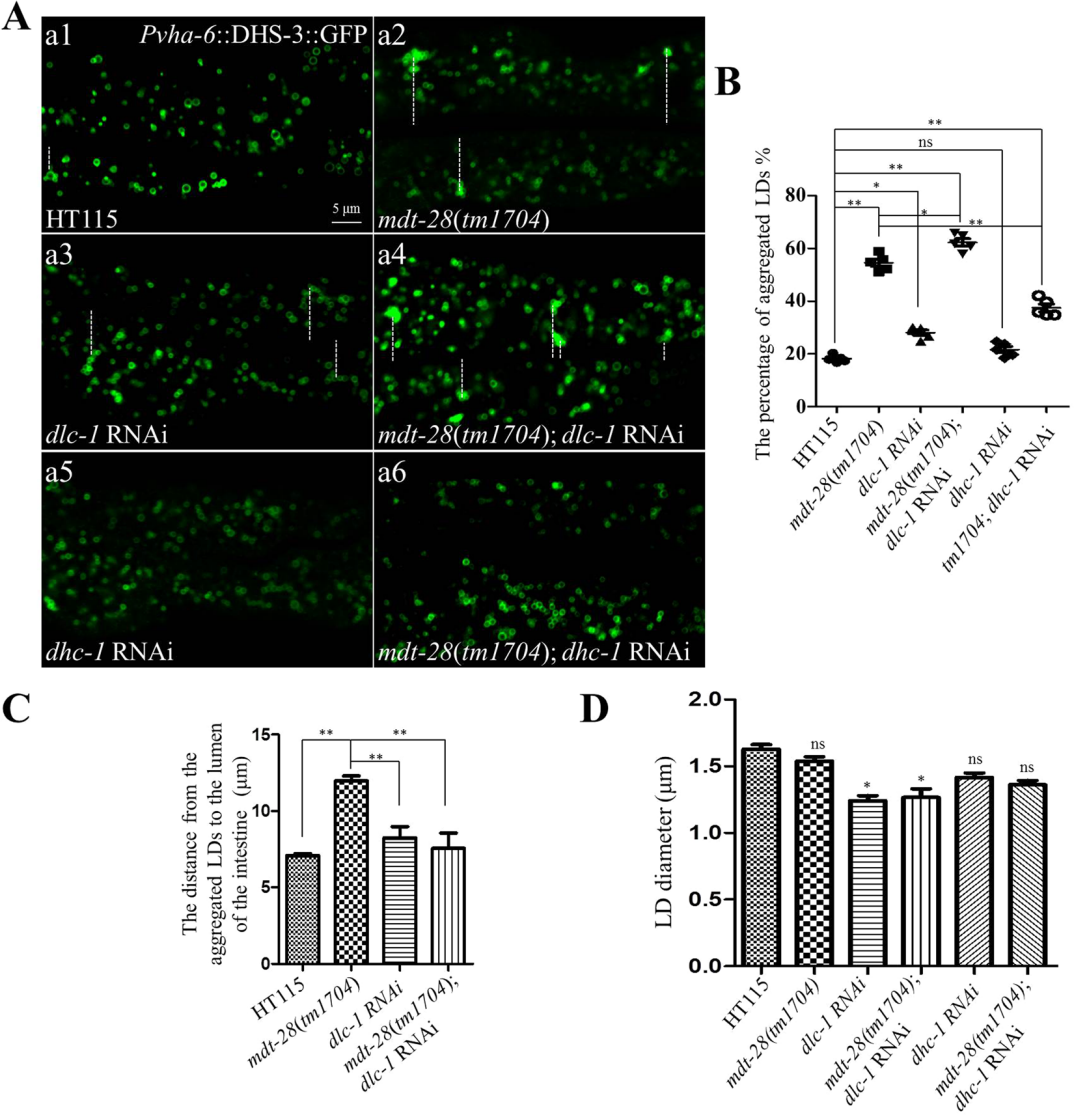
Microtubules affect the morphology of LDs. (**A**) Images of LDs using the marker *Pvha-6*::DHS-3::GFP in a larval L4 stage animal (a1), *Pvha-6*::DHS-3::GFP; *mdt-28*(*tm1704*) worms (a2), *Pvha-6*::DHS-3::GFP; *dlc-1* RNAi worms (a3), *Pvha-6*::DHS-3::GFP; *mdt-28*(*tm1704*); *dlc-1* RNAi worms (a4), *Pvha-6*::DHS-3::GFP; *dhc-1* RNAi worms (a5), *Pvha-6*::DHS-3::GFP; *mdt-28*(*tm1704*); *dhc-1* RNAi worms (a6) are shown, Scale Bar, 5 μm. The white line represents the distance from the aggregated LDs to intestinal axis. (**B**) The percentage of aggregated LDs as scored visually (A). Data represent mean ± SEM (n = 5 for each independent experiment, **P < 0.01, 0.01 < *P < 0.05, ns, no significance, two-way ANOVA). (**C**) The distance from the aggregated LDs to intestinal axis was quantified (Aa1-a4). Data represent mean ± SEM (n = 5 for each independent experiment, **P < 0.01, two-way ANOVA). (**D**) Quantification of the LD diameter (A). Data represent mean ± SEM (n = 5 for each independent experiment, 0.01 < *P < 0.05, ns, no significance, two-way ANOVA).

Since DLC-1 interacts with MDT-28/PLIN-1, we investigated the influence of DLC-1 on the LD phenotype in wild type worms and *mdt-28* mutants. We previously found that LDs were uniformly dispersed in the cytoplasm of *Pvha-6::dhs-3::GFP* worms. In contrast, in intestinal cells, about 57% of LDs in *mdt-28*(*tm1704*) mutants became aggregated and the aggregated LDs located at the edge of the cells (Fig. 5Aa1,Aa2,B,C).

Knock down of *dlc-1* in wild type worms resulted in slow growth and poor health. Approximately 24.8% of the LDs in these animals were aggregated, but the aggregated LDs were scattered and distributed in a disorderly fashion in intestinal cells (Fig. 5Aa3,C). Knock down of *dlc-1* in *mdt-28* mutants slightly increased the percentage of aggregated LDs to 63% (Fig. 5Aa4,C), and the aggregated LDs were also scattered in the intestinal cells. However, knockdown of *dlc-1* partially restored aggregated LD distribution in *mdt-28*(*tm1704*). Therefore, it is possible that DLC-1 affected LD distribution partially through MDT-28/PLIN-1.

Next, we knocked down *dhc-1* which has a function similar to *dlc-1* in the microtubule transportation process. In wild type animals, the percentage of aggregated LDs after *dhc-1* knock down was 23.7% (Fig. 5Aa5,B). The degree of aggregation was 35.8% after *dhc-1* knockdown in *mdt-28*(*tm1704*) animals (Fig. 5Aa6,B). This result demonstrates that DHC-1 may have a function similar to DLC-1 in the regulation of LDs distribution. Interestingly, knock down of either *dlc-1* or *dhc-1* in the wild type resulted in reduced LD size but had no significant effect in the *mdt-28*(*tm1704*) mutant (Fig. 5D). Therefore, microtubules influence LD distribution, in part through MDT-28/PLIN-1.

### Model for MDT-28/PLIN-1 function in *C*. *elegans*

**Figure 6:**
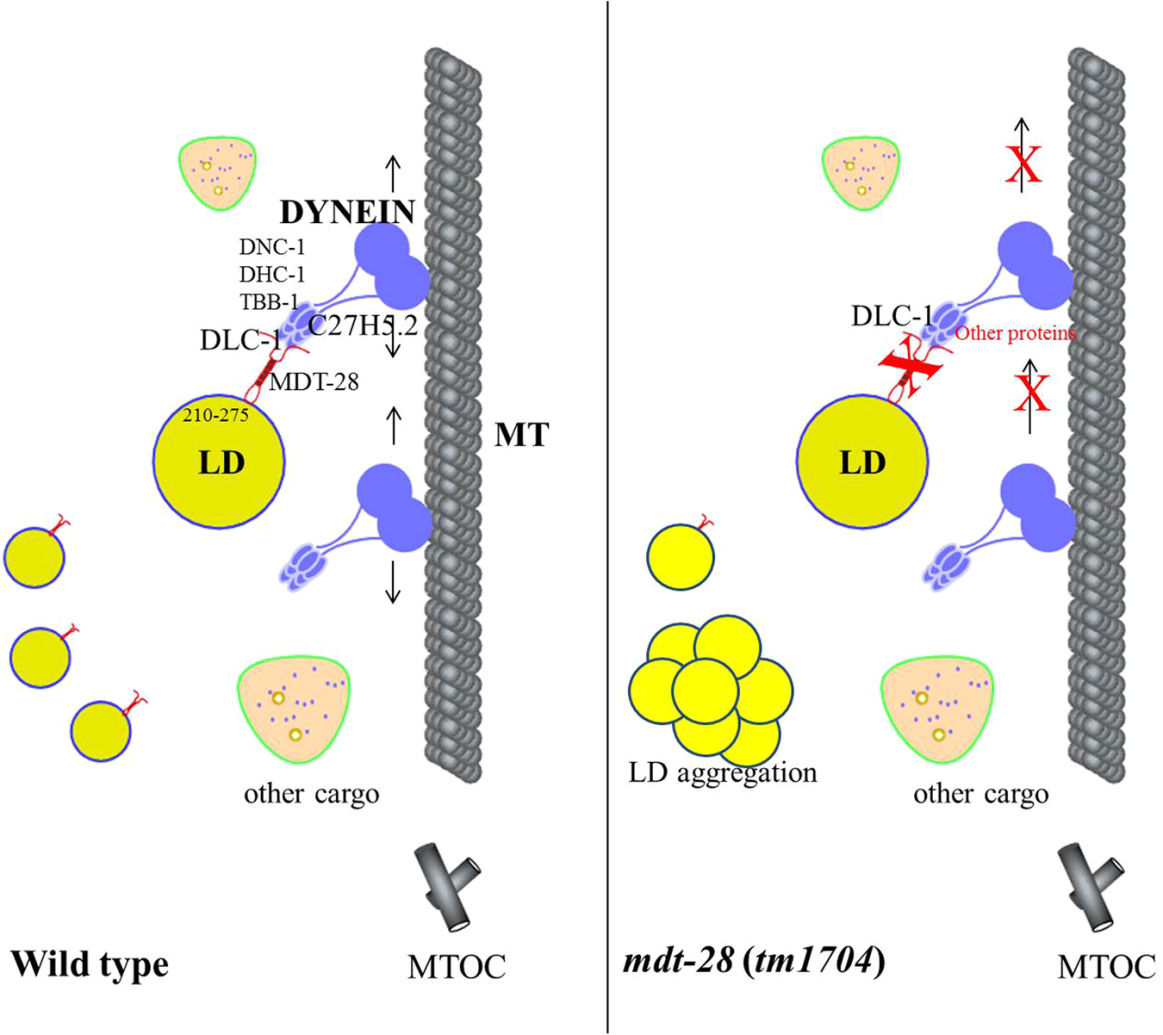
Model for MDT-28 function in *C*. *elegans*. Model of the molecular mechanism controlling LD association with cytoskeleton. In the wild type, on the LD membrane, MDT-28/PLIN-1 functions as an adaptor protein for dynein, a molecular motor. MDT-28/PLIN-1 directly binds to DLC-1 through its amino acids 1–210 and 275–415, similar to two hands grasping the microtubules. In contrast, Deletion of MDT-28/PLIN-1 leads to the aggregated LDs in intestinal cells.

Our results provide evidence that MDT-28/PLIN-1, the most abundant LD protein in *C*. *elegans*, plays a key role in LD distribution. Here we propose a working model for how MDT-28/PLIN-1 mediates LD distribution in *C*. *elegans* (Fig. 6). MDT-28/PLIN-1 directly binds DLC-1 through amino acids 1–210 and 275–415. The deletion of *mdt-28* partially prevents microtubule-dependent movement of LDs, resulting in the LD aggregation. Similarly, suppression of *mdt-28*, *dhc-1*, or *dlc-1* impairs movement of LDs along microtubules, giving rise to an increased percentage of aggregated LDs.

## Discussion

Lipid droplet dynamics are linked to many metabolic diseases^4–6^. Lipid droplet distribution in cells is crucial for lipid utilization in *C*. *elegans*. In this study, we used genetic, live imaging, and biochemical methods to demonstrate that MDT-28/PLIN-1 is involved in the distribution and the morphology of LDs in *C*. *elegans*. As the most abundant LD protein^13^, we showed that MDT-28/PLIN-1 is involved in the regulation of LD distribution along microtubules by directly binding with DLC-1.

For the phenotype of *dlc-1* RNAi; *mdt-28*(*tm1704*), knockdown of *dlc-1* in *mdt-28* mutants slightly increased the percentage of aggregated LDs, but *dlc-1* RNAi partially restored aggregated LD distribution in *mdt-28*(*tm1704*). We suspect that there may be some proteins on the LDs that interact with the microtubules, and MDT-28 may be just one of them.

In the present work, mutations of *C27H5*.*2* and *mdt-28* induced similar lipid droplet phenotypes, and further studies implicated DLC-1 as an MDT-28/PLIN-1 interacting protein probably playing an important role in the LD aggregation phenotype. A previous study showed that C27H5.2 interacts with UNC-83 by yeast two-hybrid assay, suggesting a possible interaction between UNC-83 and DLC-1^35^. Thus, DLC-1, UNC-83, and C27H5.2 may be dynein-mediated complexes involved in LD distribution. However, in this model, we only identified MDT-28/PLIN-1 as an DLC-1 interacting protein. A possible explanation for the lack of detectable interaction between UNC-83 and DLC-1 may be due to the developmental stage examined in this study. UNC-83 is important during early developmental stages of *C*. *elegans*. An alternative explanation is that the study did not focus on LD-associated proteins.

MDT-28/PLIN-1 prevents LDs from aggregating in *C*. *elegans* and thus plays a role similar to ADRP in mammals^38^. An analysis of the MDT-28/PLIN-1 sequence reveals that this protein shares sequence similarity to the transcriptional mediator complex (MED28). It also contains an N-terminal domain, part of which is conserved in the perilipin family^25^. The PAT domain in the 1–210 sequences of MDT-28/PLIN-1 suggests that the perilipin family proteins could interact with DYNLL (DLC-1 homologous protein) in mammals.

In summary, our results reveal that DHS-3 can be used as a marker protein to study LD distribution such as the aggregation phenotype in live *C*. *elegans*. Also, we identified DLC-1 as a binding partner of MDT-28/PLIN-1 which may be involved in regulating LD interaction with microtubules. We reported for the first time the involvement of MDT-28/PLIN-1, a major LD scaffold protein, in the complex interactions between LDs and microtubules.

## Materials and Methods

### Nematode strains and RNAi constructs

All *C*. *elegans* strains were handled and maintained following standard procedures^39^. The N2 Bristol strain was used as wild type in this study. *CB4856*^40^ was obtained from the *Caenorhabditis* Genetics Center. LIU5 {*hjSi224* [*vha-6p::dhs-3::gfp*]} was generated by professor Ho Yi Mak’s laboratory. *sEx10571* [*rCesT26A5*.*9*::GFP + *pCeh361*] was provided by professor Xiaochen Wang. LIU210 {*adIs2122* [*lgg-1p::GFP::lgg-1* + *rol-6* (*su1006*)]} was provided by professor Hong Zhang. LIU192 {*OLS400 aarSi1* [*dlc-1p::dlc- 1::gfp*, *unc-119*(+)]} was provided by Anders Olsen’s laboratory. LIU215 {*CasIs581 [dlc-1::GFP ki*]}^37^ was provided by Guangshuo Ou’s laboratory. The LIU102 strain {*ldrEx80 [dlc-1p::dlc-1::GFP*, *odr-1p::dsRed*]} was constructed by in our laboratory. The LIU2 {*ldrIs2 [mdt-28p::mdt-28::mCherry*, *unc-76*(+)]}strains was previously described^13^. The extrachromosomal array was integrated by UV irradiation using standard methods^41^ to generate LIU2 *ldIs2* [*mdt-28p::mdt-28::mCherry* + unc-119(+)]. Integrated lines were outcrossed three times to N2 wild type animals.

### RNAi assay of *C*. *elegans*

HT115 was used as the control for the RNAi assay. The RNAi for *dlc-1* and other genes were from the Ahringer RNAi library^42^. The synchronized L1 worms were cultured on the RNAi NGM plates at 20 °C to generate F1 worms for phenotypic analysis.

### Forward genetic screen and mutant mapping

Mutants were obtained by a forward genetic screen using Ethyl methane sulfonate (EMS) as previously described^43,44^. We screened ~15,000 haploid genomes and obtained 140 mutants. Four major phenotypes were determined as: 1) ‘enlarged’ phenotype, based on the size of LDs above 3 μm, 2) the phenotype ‘aggregated’, with at least 5 LDs clustered together, 3) the phenotype ‘aggregated and small’, with at least 5 LDs clustered with LD sizes <1.0 μm, and 4) the phenotype ‘decreased’ with a reduced number of LDs (Compared with the wild type, the number is significantly reduced, reduced more than 50%). Before mapping, mutant strains were outcrossed to N2 6 times to remove the background, and those with stable phenotypes were outcrossed to CB4856 and 1,000 F2 worms were singled out from the resulting progeny. The selected F2 worms were lysed using lysis buffer following standard procedures, and the lysis product (genomic DNA) was used as the polymerase chain reaction (PCR) template for single-nucleotide polymorphism (SNP) mapping^40^. Briefly, when the mutation sites were mapped to a small range in the chromosome, the genes in this region were knocked down one by one. If a knock down of the candidate gene in the wild type led to the expected phenotype while a subsequent knock down of the gene in the related mutant yielded no significant difference in phenotype, we concluded that the gene was responsible for the phenotype.

### Drug preparation

The compound Nocodazole (CAS: 31430-18-9) was dissolved in DMSO. The Nocodazole was added in the NGM medium at a final concentration of 5 µM. Synchronized L1 worms were placed onto the NGM plates supplemented with or without Nocodazole, and the larval L4 stage worms were picked for further analysis.

### Yeast two-hybrid assay

A yeast two-hybrid screen was carried out according to the methods described in the Yeastmaker Yeast Transformation System 2 User Manual. The inserts containing DNA sequences encoding MDT-28/PLIN-1 was cloned into the vectors pPC97. Then the pPC97-*mdt-28* plasmid was transformed into yeast *ma203* (wild type), the transformants were grown on synthetically defined SD/-Trp-Leu dropout medium plates for 3–5 days. After pPC97-*mdt-28 yeast was obtained*, 10 µg *C*. *elegans*-cDNA library was transformed into the yeast strain pPC97-*mdt-28*. The yeast cells with co-transformation of the 2 fusion plasmids were further dropped on SD/-Trp-Leu-His-Ade dropout medium plates with a series of 10-fold dilutions for checking the interactions. After sequencing, Hits were verified by co-transforming the pPC86-candidate gene plasmid and pPC97-*mdt-28* plasmid into yeast *ma203* (wild type), using SD/-Leu/-Trp plates to screen the positive clones. All of the clones were re-streaked and tested for activation of the lacZ reporter gene by X-gal colony-lift filter assays (Clontech). The yeast clones were transferred to and grown on Nytran N followed by lysis with liquid nitrogen. The membranes were then placed in staining buffer (60 mM Na_2_HPO_4_.7H_2_O, 40 mM NaH_2_PO_4_^.^H_2_O, 10 mM KCl, 1 mM MgSO_4_^.^7H_2_O, 20 mg/mL X-gal, 5 µL/mL 2-Mercaptoethanol) for 10 min, and were then incubated at 30 °C for 30 min. After the incubation period, the color of the clones was examined.

### *In vitro* pull-down assays

To examine the interaction between MDT-28/PLIN-1 and DLC-1, *E*. *coli* strain (BL21) was used to express GST, GST-MDT-28 (1–210, 210–275, 275–415, full length) and His-tagged truncations of DLC-1 and LGG-1 (). Bacterial cultures were disrupted in the lysis buffer [25 mM Tris-HCl (pH 7.5), Nonidet P40, 150 mM NaCl, 0.5% 10% glycerol]. The cleared supernatants were mixed and incubated with Glutathione Sepharose 4B beads (GE HealthCare, 170756) at 4 °C for 4–6 hr. After three times wash with the lysis buffer, the bounded proteins were eluted with 1 X SDS loading buffer. The pull-down product was detected by Western blotting with anti-His antibody.

### Fluorescence imaging of *C*. *elegans*

Fluorescence images of L4 larval animals were obtained using a laser scanning confocal microscope (LSM 710 Meta, ZEISS). The 3D images were obtained using a laser scanning confocal microscope (LSM 880 Meta, ZEISS) with airyscan. Images were processed and viewed using ZEN 2011 software (ZEISS).

### Brood size analysis

Approximately 20 L4 worms were removed from synchronized mothers fed with OP50 and were transferred to NGM plates seeded with the appropriate bacteria, in triplicate. The worms were transferred to new plates each day until no additional embryos were produced. The number of embryos was counted each day^45,46^.

### Quantification of aggregated lipid droplets

Micrographs of the L4 stage worms were prepared. The percentage of aggregated LDs was calculated as the ratio of aggregated LDs/total LD (in the tail of the nematode). A cluster that had more than 10 LDs was counted as aggregated (no distance between LDs and LDs) in three-dimensional (3D) images. A cluster that had more than 5 LDs was counted as aggregated LDs in two-dimensional (2D) images. For the 2D imaging results, the maximum surface of the aggregated LDs layer in the 3D imaging process is selected to show this phenotype. ZESIS and Image J software was used to quantitatively analyze data.

### Data analysis

All numerical data were plotted as mean ± SEM. LD size and the percentage of aggregated LDs was quantified using ImageJ and GraphPad Prism 5 (NIH, USA). Determination of significance between groups was performed using Student *t*-tests, or Two-way ANOVA, as indicated. At least five worms of each phenotype were examined for each experiment.

## Supplementary information

Supplementary file

Supplementary Information

Supplementary Information
