# Supplementary material for: MDT-28/PLIN-1 mediates lipid droplet-microtubule interaction via DLC-1 in Caenorhabditis elegans
Source: Sci Rep. 2019 Oct 17;9:14902. doi: 10.1038/s41598-019-51399-z (PMC6797801; doi:10.1038/s41598-019-51399-z)
Supplement: Supplementary file 1 — Supplementary file [file 41598_2019_51399_MOESM1_ESM.docx]

**Original data:**

**Figure 4B**

Pull down.

Samples were loaded in order as shown in figure. HIS-DLC-1 input was diluted 1:30 with 2 x sample buffer. (The primary His elution solution was 500 μl in total) GST-MDT-28 input 1:5 diluted with 2 x sample buffer. (The primary GST-MDT-28 binding beads was 500 μl in total) GST control beads 1μl for reaction, dissolved in 40 μl sample buffer, 1:3 dilute For WB and this staining, GST-MDT-28 beads 10 μl reacted with 20 μl His elution, dissolved in 40 μl sample buffer, loaded directly.

Pull down

Samples were loaded in order as shown in figure. HIS-DLC-1 input was diluted 1:30 with 2 x sample buffer. (The primary His elution solution was 500 μl in total) GST-MDT-28 input 1:5 diluted with 2 x sample buffer. (The primary GST-MDT-28 binding beads was 500 μl in total) GST control beads 1 μl for reaction, dissolved in 40 μl sample buffer, 1:3 dilute for WB and this staining, GST-MDT-28 beads 10 μl reacted with 20 μl His elution, dissolved in 40 μl sample buffer, loaded directly.

**Figure 4C**


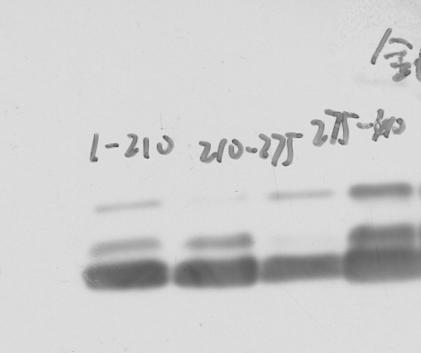


Samples were loaded in order as shown in figure. MDT-28 (1-210)-GST, MDT-28 (210-275)-GST, MDT-28 (275-415)-GST, MDT-28 (Full length)-GST. His-DLC-1 binding by MDT-28 (1-210, 211-274, 275-415)-GST fusion proteins was detected by Western blot with anti-His.
