# Supplementary material for: MDT-28/PLIN-1 mediates lipid droplet-microtubule interaction via DLC-1 in Caenorhabditis elegans
Source: Sci Rep. 2019 Oct 17;9:14902. doi: 10.1038/s41598-019-51399-z (PMC6797801; doi:10.1038/s41598-019-51399-z)
Supplement: Supplementary file 2 — Supplementary Information [file 41598_2019_51399_MOESM2_ESM.pdf]

MDT-28/ PLIN-1 mediates lipid droplet-microtubule interaction via DLC-1 in *Caenorhabditis elegans*

Kang Xie[1,2#], Peng Zhang[3,4#], Huimin Na[5], Yangli Liu[1,2], Hong Zhang[1,2*], and Pingsheng Liu[1,2*]
[1]National Laboratory of Biomacromolecules, CAS Center for Excellence in Biomacromolecules, Institute of Biophysics, Chinese Academy of Sciences, Beijing 100101, China
[2]University of Chinese Academy of Sciences, Beijing 100049, China
[3]Research Division, Joslin Diabetes Center, One Joslin Place, Boston, MA 02215, USA
[4]Department of Genetics and Harvard Stem Cell Institute, Harvard Medical School, Boston, MA 02215, USA
[5]Program in Systems Biology and Program in Molecular Medicine, University of Massachusetts Medical School, Worcester, MA, USA
[#]These authors contributed equally to this work.
[*]Corresponding author: pliu@ibp.ac.cn (Pingsheng Liu)

hongzhang@ibp.ac.cn (Hong Zhang)

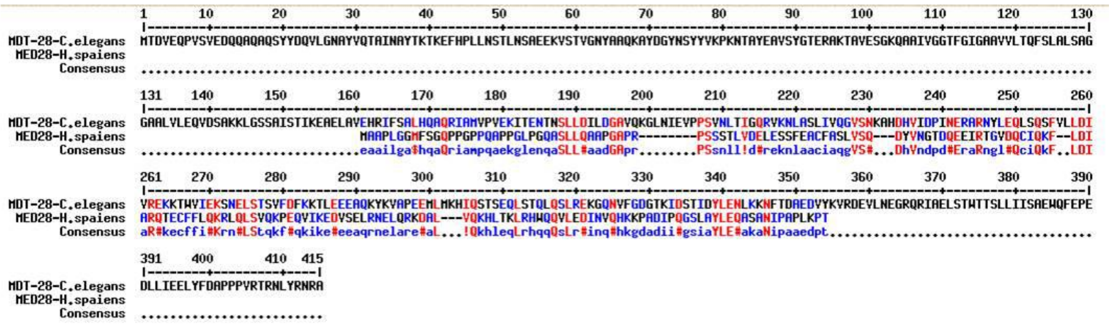

Supplemental Figure S1. MED28 is the homologous protein of MDT-28/PLIN-1**.**

Alignment of MDT-28, MED28 using http://multalin.toulouse.inra.fr/multalin/multalin.html.

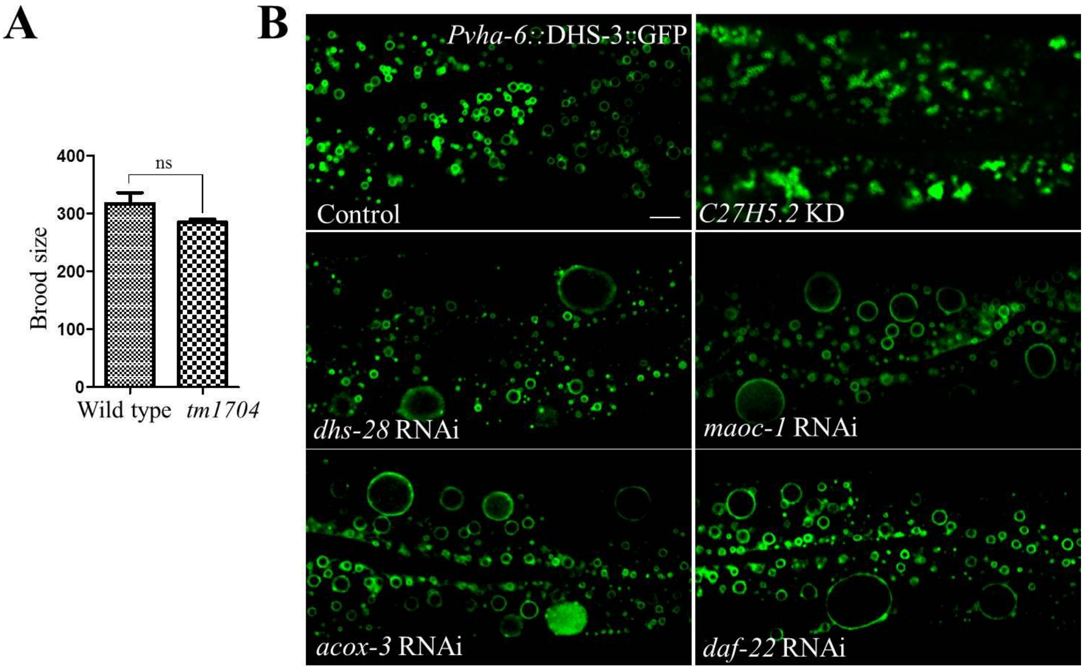

Supplemental Figure S2. Knock down of EMS-related genes in WT gives rise to EMS-related mutant phenotype.

(A) Brood sizes of wild type and *mdt-28*(*tm1704*) worms were determined at 20°C, Data represent mean ± SEM (n=5 for each independent experiment, ns, no significance, P>0.05, student *t*-test).

(B) Knock down of *maoc-1, dhs-28, daf-22, acox-3,* and *C27H5.2* in *Pvha-6::*DHS-3::GFP worms. Scale Bar, 5 µm.

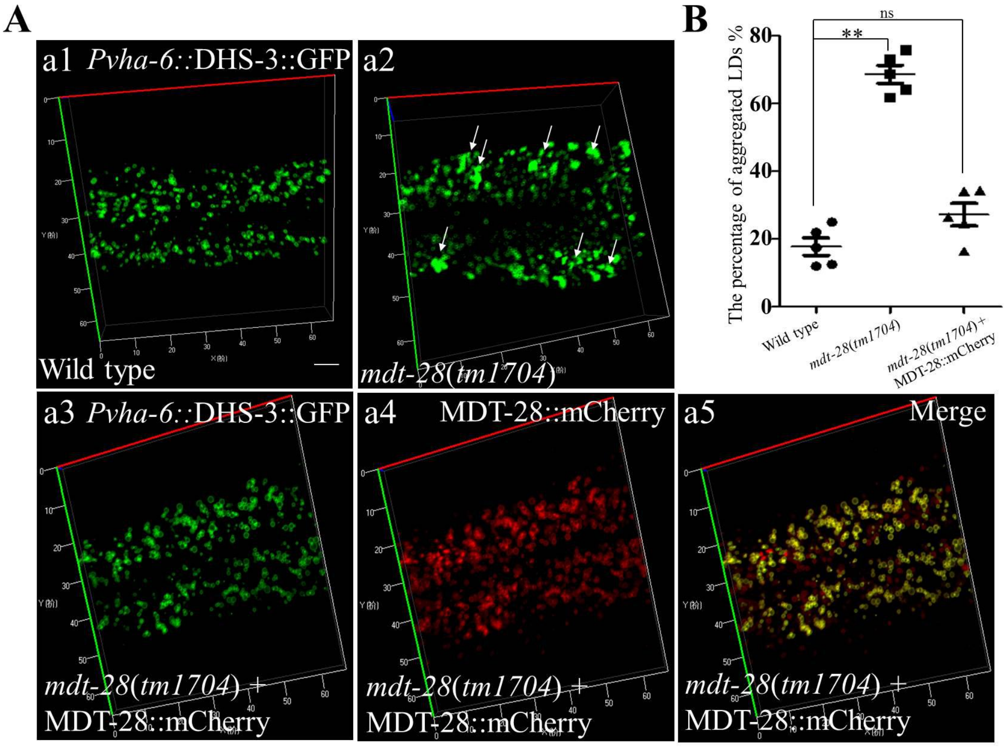

Supplemental Figure S3. Mutation of *mdt-28* leads to aggregated lipid droplets.

(A) Fluorescence 3D micrographs of LDs in the intestine. Scale Bar, 5 µm. (**a1**) Fluorescence 3D micrographs of *Pvha-6*::DHS-3::GFP in a larval L4 stage animal. (**a2**) Visualization of LDs using the marker *Pvha-6*::DHS-3::GFP in the *mdt-28*(*tm1704*) mutant. The white arrows point to aggregated LDs. (**a3, a4, and a5**) As in (a2), but with the *mdt-28*(*tm1704*) mutant animal carrying a recue transgene [*mdt-28p::mdt-28::mCherry*]. **(B)** Quantification of the percentage of aggregated LDs (A). Data represent mean ± SEM (n=5 for each independent experiment, **P<0.01, ns, no significance, one-way ANOVA).

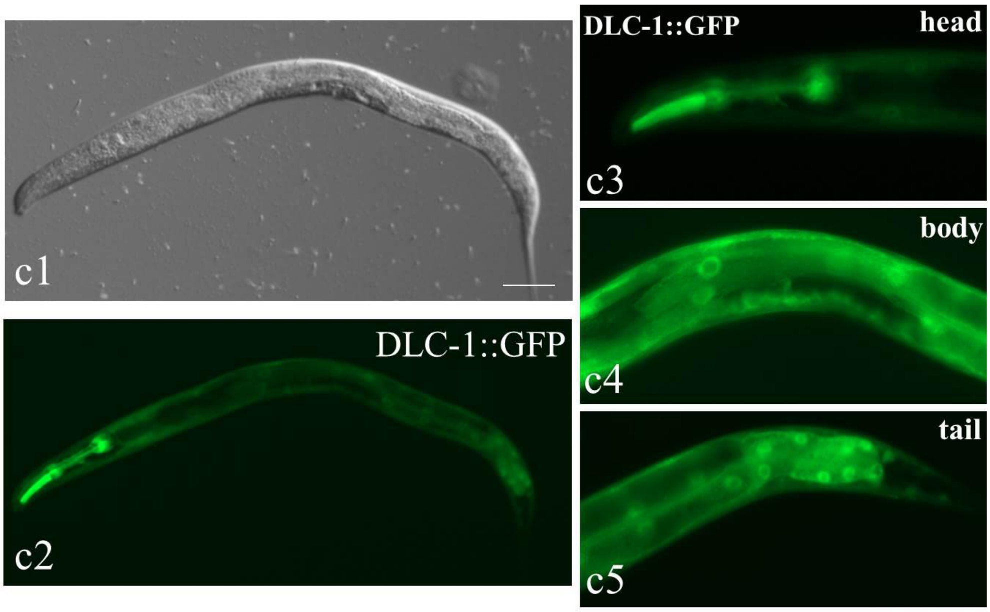

Supplemental Figure S4. The expression pattern of DLC-1::GFP.

(**c1**) A bright field image of the expression of DLC-1::GFP under control of the *dlc-1* promoter in L4 stage animals. Scale Bar, 50 μm. (**c2**) Visualization of DLC-1::GFP expression in the whole worm. (**c3**) DLC-1::GFP is expressed in adult pharynx and unidentified cells in the head. (**c4**) DLC-1::GFP is expressed in the intestine, germ cells and oocytes. (**c5**) DLC-1::GFP expression in body wall muscle and posterior intestine.

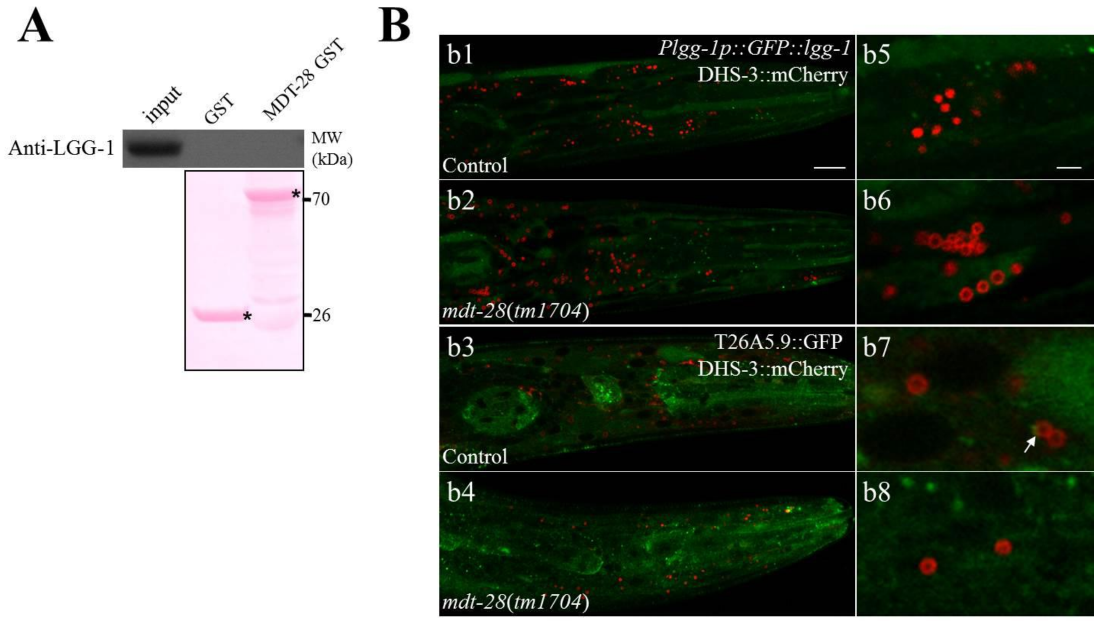

Supplemental Figure S5. LGG-1 is not bound to MDT-28/PLIN-1.

(A) In a pull-down assay, MDT-28-GST immobilized on glutathione Sepharose beads did not bind His-LGG-1 as detected by Western blot with anti-LGG-1. **(B)** Images of DHS-3::mCherry labeled LDs (red) interacting with LGG-1 or DLC-1 (green). (**b1**) Visualization of GFP::LGG-1 in the wild type larval L4 stage animal. The LD marker DHS-3::mCherry was used and merged with GFP::LGG-1. Scale Bar, 10 µm. (**b2**) As in (b1), but in a *mdt-28*(*tm1704*) mutant. (**b3**) Visualization of DHC-1::GFP in the wild type larval L4 stage animal. The LD marker DHS-3::mCherry was used and merged with DHC-1::GFP. (**b4**) As in (b3), but in a *mdt-28*(*tm1704*) mutant. (**b5, b6, b7, b8**) are the enlarged pictures of (b1, b2, b3, b4), the white arrows point to the co-localization sites. Scale Bar, 2 µm.

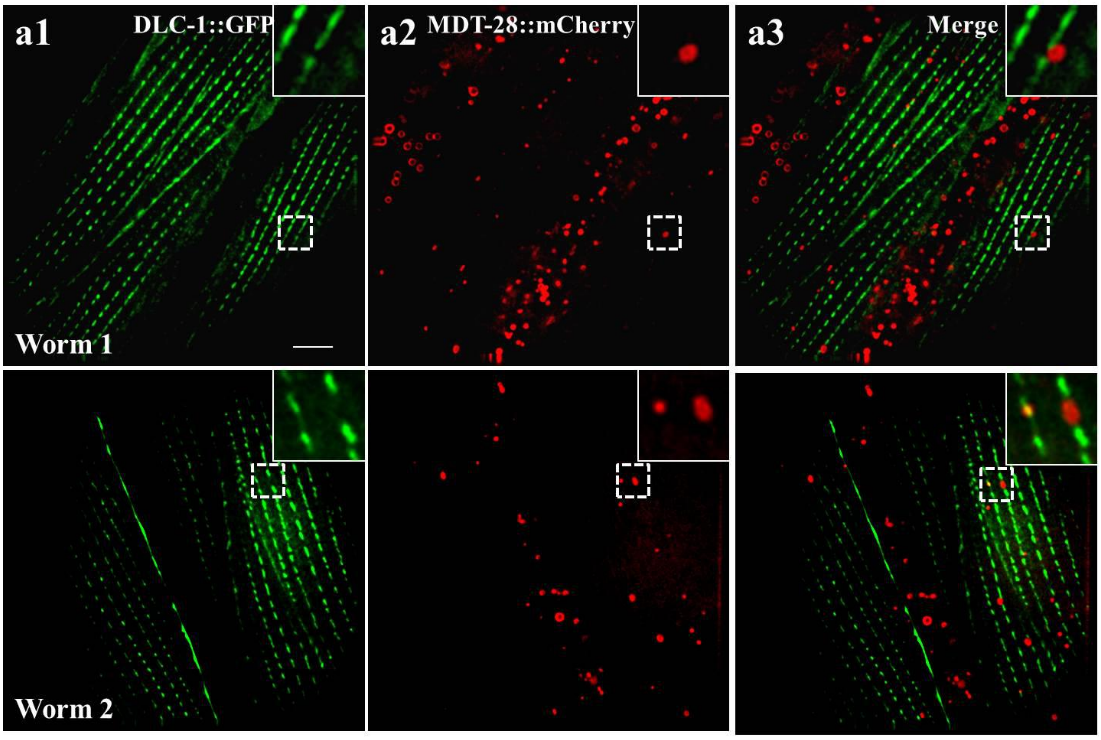

Supplemental Figure S6. DLC-1 is bound to MDT-28/PLIN-1.

(**A**) SIM images of LDs (red) interacting with DLC-1 (green). Scale Bar = 5 μm. (**a1**) Visualization of microtubules in the body wall muscle using the marker protein DLC-1::GFP. (**a2**) Visualization of LDs in the body wall muscle using the marker MDT-28::mCherry. (**a3**) as in (a1), but with gfp and mCherry signals merged.

Supplemental Table S1 List of the microtubule, cytoskeleton, actin, and filament related genes.

Table S1 listed the genes examined and those displaying LD phenotypes. RNAi clones were from Ahringer RNAi library. "N" represents no statistically significant difference compared with wild type. For the blank results in the table, no useful bacteria in the RNAi library.
