# Supplementary material for: MDT-28/PLIN-1 mediates lipid droplet-microtubule interaction via DLC-1 in Caenorhabditis elegans
Source: Sci Rep. 2019 Oct 17;9:14902. doi: 10.1038/s41598-019-51399-z (PMC6797801; doi:10.1038/s41598-019-51399-z)
Supplement: Supplementary file 3 — Supplementary Information [file 41598_2019_51399_MOESM3_ESM.pdf]

Gene Name	Sequence Name	Plate	Well	Chromosome	LD phenotype	Growth status
rmd-1	*T05G5.7*	82	H11	III	slightly cluster	N
rmd-6	*R13H9.1*	98	E11	IV	small and less	slow 1 day
sup-35	*Y48A6C.3*	85	E12	III	N	slow 2 days
C16C8.18	*C16C8.18*	40	A03	II	small and less	N
rmd-5	*T23B3.3*	10	B05	I	small and less	slow 2 days
pfd-2	*H20J04.5*	42	B10	II	N	N
pfd-5	*R151.9*	77	E03	III	less	slow 2 days
maph-1.1	*F32A7.5*	26	F01	I	slightly cluster	slow 2 days
ebp-3	*Y59A8B.9*					
ZC262.7	*ZC262.7*					
spas-1	*C24B5.2*	147	A03	V	small	slow 2 days
pfd-1	*C08F8.1*	111	G09	IV	small and less	slow 1 day
pfd-6	*F21C3.5*	11	E01	I	small and less	N
rmd-3	*B0491.3*	58	B01	II	small and less	slow 2 days
D2096.11	*D2096.11*	105	B10	IV	N	N
maph-1.2	*F25D7.4*	17	H08	I	N	slow 2 days
maph-1.3	*C36A4.5*	69	G04	III	N	slow 2 days
klp-15	*M01E11.6*	7	C08	I	small and less	slow 2 days
rmd-4	*F36H12.11*	99	A09	IV	slightly cluster	N
ska-1	*Y106G6H.15*	18	A11	I	N	slow 2 days
klp-7	*K11D9.1*	85	A12	III	N	N
pfd-3	*T06G6.9*	23	A03	I	N	slow 2 days
H13N06.7	*H13N06.7*					
tac-1	*Y54E2A.3*	64	B10	II	N	slow 2 days
klp-13	*F22F4.3*	185	B07	X	small and less	N
cls-2	*R107.6*	81	E05	III	small and less	N
lgg-2	*ZK593.6*	111	C01	IV	small and less	N
rmd-2	*C27H6.4*	148	H05	V	small and less	N
ptrn-1	*F35B3.5*	202	F02	X	less	N
ptl-1	*F42G9.9*	67	A12	III	N	slow 2 days
T08G11.3	*T08G11.3*	14	G05	I	small and less	slow 1 day
txdc-9	*C05D11.3*	75	F11	III	small and less	slow 1 day
rod-1	*F55G1.4*	103	A06	IV	small and less	slow 1 day
H03A11.2	*H03A11.2*	200	C03	X	smaller and less	slow 3 days
san-1	*ZC328.4*	9	D01	I	N	slow 2 days
evl-20	*F22B5.1*	51	F12	II	slightly cluster	N
maph-9	*C34D4.1*	102	B01	IV	small and less	N
zyg-9	*F22B5.7*	51	G06	II	N	N
zyg-8	*Y79H2A.11*					N
C14H10.2	*C14H10.2*	192	B09	X	N	slow 2 days
ebp-1	*Y59A8B.7*					N
knl-1	*C02F5.1*	79	E06	III	N	slow 2 days
clp-2	*T04A8.16*	71	F06	III	slightly cluster	N
tbg-1	*F58A4.8*	82	F10	III	smaller and less	slow 2 days
cls-1	*C07H6.3*	78	A07	III	N	N
tba-1	*F26E4.8*	16	F03	I	smaller and less	slow 1 days
lis-1	*T03F6.5*	88	F04	III	smaller and less	slow 1 days

unc-116	*R05D3.7*	79	H08	III	N	N
szy-20	*C18E9.3*	53	A03	II	cluster	N
cls-3	*ZC84.3*	81	G10	III	smaller and less	slow 2 days
F56C9.10	*F56C9.10*	77	G04	III	smaller and less	N
mel-26	*ZK858.4*	15	B09	I	slightly cluster	slow 2 days
F33H2.6	*F33H2.6*	26	G10	I	smaller and less	slow 2 days
tbb-2	*C36E8.5*	70	A08	III	smaller and less	slow 2 days
csn-3	*Y38C1AA.2*					
cct-3	*F54A3.3*					
tbb-4	*B0272.1*	190	H05	X	smaller	slow 1 day
ndc-80	*W01B6.9*	109	B08	IV	N	slow 2 days
dnc-1	*ZK593.5*	111	B12	IV	slightly cluster	N
bmk-1	*F23B12.8*	159	A12	V	N	N
dcn-1	*H38K22.2*	70	F07	III	smaller	slow 2 days
saps-1	*C47G2.5*	58	A04	II	smaller	slow 2 days
Y66D12A.10	*Y66D12A.10*					
ebp-2	*VW02B12L.3*	58	C05	II	N	N
mec-12	*C44B11.3*	68	G09	III	N	N
tbcd-1	*F16D3.4*	13	E04	I	smaller and less	slow 2 days
let-711	*F57B9.2*	76	F07	III	smaller and less	slow 2 days
mei-1	*T01G9.5*	13	D03	I	N	N
nab-1	*C43E11.6*	4	E08	I	smaller	slow 2 days
klp-12	*T01G1.1*	112	D02	IV	N	N
klp-11	*F20C5.2*	105	H07	IV	N	N
klp-19	*Y43F4B.6*	88	D12	III	N	N
zen-4	*M03D4.1*	100	E04	IV	N	N
elp-1	*F38A6.2*	173	H02	V	slightly cluster	slow 1 day
zyg-12	*ZK546.1*	43	E07	II	N	N
spd-2	*F32H2.3*	14	H04	I	smaller and less	N
lin-5	*T09A5.10*	50	E05	II	slightly cluster	slow 3 days
clip-1	*M01A8.2*	81	H09	III	N	slow 2 days
cul-3	*Y108G3AL.1*					
dhc-3	*B0365.7*	155	H10	V	N	slow 2 days
unc-33	*Y37E11C.1*	95	E04	IV	N	N
tag-241	*C34E11.3*	194	F07	X	smaller and less	slow 1 day
gip-1	*H04J21.3*	68	C03	III	N	N
noca-1	*T09E8.1*	156	A01	V	smaller and less	slow 1 day
dhc-1	*T21E12.4*	4	H02	I	ghtly cluster andsma	slow 1 day
ppfr-1	*F16A11.3*					
dhc-4	*W05B2.4*	85	C12	III	smaller	slow 2 days

Gene Name	Sequence Name	Plate	Well	Chromosome	LD phenotype	Growth status
Y19D2B.1	*Y19D2B.1*	57	D06	II	N	slow 1 day
tbb-6	*T04H1.9*	153	H12	V	N	N
ifd-2	*F25E2.4*	176	G01	X	slightly cluster	N
mlst-8	*C10H11.8*	5	E03	I	N	N
arf-1.2	*B0336.2*	74	A07	III	N	N
cap-2	*M106.5*	57	C02	II	N	N
add-1	*F39C12.2*	183	D07	X	N	N
exc-9	*F20D12.5*	104	C05	IV		
hil-1	*C30G7.1*	160	B01	V	N	N
tba-7	*T28D6.2*	86	A01	III	N	N
ifp-1	*C43C3.1*	191	C06	X	N	N
frm-10	*F25H9.5*	156	F03	V	N	N
plk-2	*Y71F9B.7*					
ifd-1	*R04E5.10*	190	A06	X	N	N
ifc-1	*F37B4.2*	131	D09	V		
nck-1	*ZK470.5*	182	C02	X		
ben-1	*C54C6.2*	69	C05	III		
exl-1	*F26H11.5*	63	F07	II	N	N
frm-5.1	*Y38C1AB.8*					
T08D2.8	*T08D2.8*	174	C07	V		
crp-1	*Y32F6B.3*	150	B03	V		
F54A3.2	*F54A3.2*					
ifa-1	*F38B2.1*	193	G03	X	small	N
frm-4	*C24A11.8*	6	G11	I	N	slow 2 day
osg-1	*R02F2.2*	73	E09	III	N	slow 1 day
glr-1	*C06E1.4*	80	D03	III	N	slow 1 day
fln-1	*Y66H1B.2*	92	E04	IV		
jac-1	*Y105C5B.21*					
unc-83	*W01A11.3*	140	A12	V		
anc-1	*ZK973.6*					
tln-1	*Y71G12B.11*					
hum-7	*F56A6.2*	1	D07	I	N	N
gei-4	*W07B3.2*	66	E07	III	small and less	slow 1 day
unc-54	*F11C3.3*	26	F06	I		
hmr-1	*W02B9.1*	19	C10	I	N	slow 1 day

Gene Name	Sequence Name	Plate	Well	Chromosome	LD phenotype	Growth status
unc-60	*C38C3.5*					
gsnl-1	*K06A4.3*	147	F06	V	N	N
frg-1	*ZK1010.3*	88	A06	III	N	slow 2 days
C46H11.3	*C46H11.3*	6	A09	I		
Y65B4A.4	*Y65B4A.4*					
tth-1	*F08F1.8*	189	D02	X	N	N
unc-78	*C04F6.4*	181	A09	X		
arp-1	*Y53F4B.22*					
arx-6	*C35D10.16*					
unc-87	*F08B6.4*	10	C10	I		
aipl-1	*K08F9.2*	160	F08	V	N	slow 2 days
F26H9.2	*F26H9.2*	15	D09	I		
cap-1	*D2024.6*	102	E04	IV	small	slow 1 days
arp-11	*C49H3.8*	104	B08	IV		
F36F2.1	*F36F2.1*	14	H11	I		
unc-94	*C06A5.7*	8	C06	I		
tnc-2	*ZK673.7*	56	D02	II		
act-1	*T04C12.6*	151	D05	V	N	slow 2 days
twf-2	*F38E9.5*	201	G04	X	N	N
swsn-6	*ZK616.4*					
arx-7	*M01B12.3*	2	G04	I		
vab-9	*T22C8.8*	52	C07	II		
C52B9.8	*C52B9.8*	182	E02	X	N	slow 1 days
arx-2	*K07C5.1*	149	G07	V	small	slow 2.5 days
arx-5	*Y37D8A.1*	87	G04	III	small	slow 2 days
act-5	*T25C8.2*	89	B04	III	N	slow 1 days
wip-1	*R144.4*	72	E07	III		
tag-243	*T04A8.4*	71	E07	III		
mlst-8	*C10H11.8*	5	E03	I		
cor-1	*R01H10.3*	84	B01	III	N	N
pat-10	*F54C1.7*	6	A05	I		
ham-3	*ZK1128.5*	83	H04	III	N	N
act-3	*T04C12.4*	151	D03	V	N	slow 2 days
arx-4	*Y6D11A.2*					
smrc-1	*C16A3.1*	75	E11	III	N	N
gpd-2	*K10B3.8*	180	E05	X	N	slow 2 days
spe-26	*R10H10.2*	110	A12	IV	N	N
sac-1	*F30A10.6*	15	H01	I		
lis-1	*T03F6.5*	88	F04	III	small	slow 1 days
saps-1	*C47G2.5*	58	A04	II		
wve-1	*R06C1.3*	21	A03	I		
nck-1	*ZK470.5*	182	C02	X	N	slow 1 days
osg-1	*R02F2.2*	73	E09	III	slightly cluster	N
alp-1	*T11B7.4*	106	B06	IV	N	N
arx-3	*Y79H2A.6*	86	G10	III	N	N
nab-1	*C43E11.6*	4	E08	I		
arp-6	*C08B11.6*	50	G08	II		

rho-1	*Y51H4A.3*	119	G12	IV		
Y50D7A.10	*Y50D7A.10*					
unc-120	*D1081.2*	13	G01	I		
mig-2	*C35C5.4*	194	B07	X		
swsn-3	*Y71H2AM.17*				N	slow 1 days
act-4	*M03F4.2*	183	E08	X	slightly cluster	slow 1 days
unc-115	*F09B9.2*	192	A05	X		
mlc-5	*T12D8.6*	89	B11	III		lethal
M03C11.8	*M03C11.8*	84	D07	III		
eps-8	*Y57G11C.24*	118	F03	IV	N	N
rac-2	*K03D3.10*	119	F12	IV	N	N
zoo-1	*Y105E8A.26*					
arx-1	*Y71F9AL.16*					
crp-1	*Y32F6B.3*	150	B03	V	N	N
W02H5.2	*W02H5.2*	130	F10	V	N	slow 2.5 days
deb-1	*ZC477.9*					
fli-1	*B0523.5*	80	F02	III		
hipr-1	*ZK370.3*	80	G03	III	N	N
pxl-1	*C28H8.6*	74	E07	III	N	N
T24B8.4	*T24B8.4*	53	B12	II		
mrck-1	*K08B12.5*	139	F03	V	N	N
par-4	*Y59A8B.14*					
add-1	*F39C12.2*	183	D07	X	N	N
vab-19	*T22D2.1*	37	C08	II		
kel-1	*C47D12.7*	58	H04	II		
viln-1	*C10H11.1*	5	D08	I		
glr-1	*C06E1.4*	80	D03	III		
ketn-1	*F54E2.3*	131	C01	V	N	slow 2 days
F10G8.8	*F10G8.8*	17	C01	I		
tag-273	*Y57G11A.1*					
zyx-1	*F42G4.3*	61	D07	II		
lev-11	*Y105E8B.1*	26	B02	I		
frm-1	*ZK270.2*	26	F08	I		
pes-7	*F09C3.1*	25	F09	I		
tln-1	*Y71G12B.11*					
vav-1	*C35B8.2*	190	F04	X	less	N
mig-10	*F10E9.6*	79	G05	III	N	slow 1 days
fozi-1	*K01B6.1*	81	H10	III	N	N
fln-1	*Y66H1B.2*	92	E04	IV	N	N
jac-1	*Y105C5B.21*					
anc-1	*ZK973.6*					
spe-15	*F47G6.4*	2	A04	I		
hum-7	*F56A6.2*	1	D07	I		
vab-10	*ZK1151.1*	20	F09	I		
hmr-1	*W02B9.1*	19	C10	I		

Gene Name	Sequence Name	Plate	Well	Chromosome	LD phenotype	Growth status
ifd-2	*F25E2.4*	176	G01	X	slightly cluster	slow 1 days
pfn-3	*K03E6.6*	177	B05	X	N	slow 2 days
pfn-2	*F35C8.6*	184	C03	X	N	N
unc-60	*C38C3.5*					
anmt-1	*B0303.2*					
F15G9.1	*F15G9.1*	191	C09	X	N	slow 1 days
ifb-2	*F10C1.7*	45	E11	II		
C04F12.8	*C04F12.8*	16	D10	I		
unc-94	*C06A5.7*	8	C06	I		
alp-1	*T11B7.4*	106	B06	IV		
hil-1	*C30G7.1*	160	B01	V	N	N
ifd-1	*R04E5.10*	190	A06	X	slightly cluster	N
ifc-1	*F37B4.2*	131	D09	V	N	slow 1 days
aipl-1	*K08F9.2*	160	F08	V	N	slow 2 days
ifa-4	*K05B2.3*	183	E04	X	N	slow 2.5 days
mua-6	*W10G6.3*	201	E05	X		lethal
R31.2	*R31.2*	153	B06	V	N	N
rho-1	*Y51H4A.3*	119	G12	IV		
ifp-1	*C43C3.1*	191	C06	X	N	N
gsnl-1	*K06A4.3*	147	F06	V		
git-1	*F14F3.2*	192	E10	X	N	N
ifa-3	*F52E10.5*	201	E10	X	N	N
ifb-1	*F10C1.2*	45	E07	II		
ifa-1	*F38B2.1*	193	G03	X	N	N
pat-3	*ZK1058.2*	69	H01	III		
unc-78	*C04F6.4*	181	A09	X		
nmy-1	*F52B10.1*	180	B05	X	slightly cluster	N
ppk-1	*F55A12.3*	6	F09	I		
myo-1	*R06C7.10*	11	D08	I		
pix-1	*K11E4.4*	197	H06	X	N	N
ifc-2	*M6.1*	176	D08	X	small	N
myo-3	*K12F2.1*	153	H02	V	small	slow 2 days
gei-4	*W07B3.2*	66	E07	III		
unc-96	*F13C5.6*	176	D06	X		
unc-45	*F30H5.1*	66	F03	III		
fln-1	*Y66H1B.2*	92	E04	IV		
mua-3	*K08E5.3*	83	H09	III	N	N
tln-1	*Y71G12B.11*					
unc-54	*F11C3.3*	26	F06	I		

RNAi clones were from Ahringer RNAi library

N represnents normal
